# Multiple endocrine neoplasia 2 in Cyprus: evidence for a founder effect

**DOI:** 10.1007/s40618-018-0841-0

**Published:** 2018-02-02

**Authors:** P. Fanis, N. Skordis, S. Frangos, G. Christopoulos, E. Spanou-Aristidou, E. Andreou, P. Manoli, M. Mavrommatis, S. Nicolaou, M. Kleanthous, M. A. Cariolou, V. Christophidou-Anastasiadou, G. A. Tanteles, L. A. Phylactou, V. Neocleous

**Affiliations:** 1 Department of Molecular Genetics, Function and Therapy, The Cyprus Institute of Neurology and Genetics, P.O. Box 23462, 1683 Nicosia, Cyprus; 2 Division of Pediatric Endocrinology, Paedi Center for Specialized Pediatrics, Nicosia, Cyprus; 3 St George’s, University of London Medical School at the University of Nicosia, Nicosia, Cyprus; 4 Nuclear Medicine Department, Bank of Cyprus Oncology Center, Nicosia, Cyprus; 5 Molecular Genetics Thalassaemia Department, The Cyprus Institute of Neurology and Genetics, Nicosia, Cyprus; 6 Department of Clinical Genetics, The Cyprus Institute of Neurology and Genetics, P.O. Box 23462, 1683 Nicosia, Cyprus; 7 Dasoupolis Endocrinology Center, Andrea Dimitriou Street Dasoupolis, Nicosia, Cyprus; 8 Department of Cardiovascular Genetics and the Laboratory of Forensic Genetics, The Cyprus Institute of Neurology and Genetics, Nicosia, Cyprus; 9 Cyprus School of Molecular Medicine, Nicosia, Cyprus; 10 Division of Pediatric Endocrinology, Makarios III Hospital, Nicosia, Cyprus; 11 Department of Clinical Genetics, Makarios III Hospital, Nicosia, Cyprus

**Keywords:** Multiple endocrine neoplasia type 2, *RET proto*-*oncogene*, Medullary thyroid carcinoma, Pheochromocytoma, Cancer

## Abstract

**Purpose:**

Multiple endocrine neoplasia type 2 (MEN2) affects patients with *RET* proto-oncogene mutations. This cohort study refers to patients who were diagnosed with familial medullary thyroid carcinoma (MTC) and underwent *RET* genetic testing in Cyprus between years 2002 and 2017.

**Methods and patients:**

Forty patients underwent *RET* testing by Sanger sequencing of exons 10–11 and 13–16. Genotyping with STR genetic markers flanking the *RET* gene along with Y-chromosome genotyping and haplogroup assignment was also performed.

**Results:**

*RET* mutations were identified in 40 patients from 11 apparently unrelated Cypriot families and two non-familial sporadic cases. Nine probands (69.2%) were heterozygous for p.Cys618Arg, one (7.7%) for p.Cys634Phe, one (7.7%) for the somatic delE632-L633 and two (15.4%) for p.Met918Thr mutations. The mean age at MTC diagnosis of patients carrying p.Cys618Arg was 36.8 ± 14.2 years. The age of pheo diagnosis ranged from 26 to 43 years and appeared simultaneously with MTC in 5/36 (13.9%) cases. The high frequency of the p.Cys618Arg mutation suggested a possible ancestral mutational event. Haplotype analysis was performed in families with and without p.Cys618Arg. Six microsatellite markers covering the *RET* gene and neighboring regions identified one core haplotype associated with all patients carrying p.Cys618Arg mutation.

**Conclusions:**

The mutation p.Cys618Arg is by far the most prevalent mutation in Cyprus followed by other reported mutations of variable clinical significance. The provided molecular evidence speculates p.Cys618Arg mutation as an ancestral mutation that has spread in Cyprus due to a possible founder effect.

## Introduction

Multiple endocrine neoplasia 2 (MEN2) is an autosomal dominant inherited cancer syndrome categorized into three clinically distinct forms known as MEN2A, MEN2B and Familial medullary thyroid carcinoma (FMTC) [1]. MEN2A is characterized by the association with medullary thyroid carcinoma (MTC) in 95%, pheochromocytoma (pheo) in 50% and parathyroid hyperplasia or adenoma in 15–30% of the cases [2]. MEN2B is the most aggressive type of the MEN2 variants and is characterized by abnormal body proportion seen as decreased upper/lower body ratio, marfanoid habitus and mucosal and intestinal ganglioneuromatosis [3].

MEN2A and MEN2B were first reported to be caused by point mutations in the *RET* proto-oncogene [4, 5]. The *RET* proto-oncogene encodes a transmembrane protein tyrosine kinase receptor involved in the transduction of signals for cell growth and differentiation in human neural crest-derived and neuronal tissues, such as Schwann cells, sympathetic ganglia, adrenal medulla, astrocytes and cerebral cortical neurons [6].

Up-to-date, more than 100 gain-of-function *RET* proto-oncogene mutations have been reported in patients with MTC, including germline mutations in patients with hereditary disease and somatic mutations in patients with sporadic disease [7]. The majority of the *RET* proto-oncogene mutations are located in exons 10, 11, 13, 14, 15, and 16 [2, 8–11]. Several germline mutations in exon 10 of the *RET* proto-oncogene in codons 609, 611, 618, 620 have also been associated in Hirschsprung disease [1, 2]. The clinical expression of MEN2 exhibits variable expression with different amino acid *RET* substitutions at the same codon. Such mutations are explicitly located in cysteines within this extracellular cysteine-rich domain and give rise to the subtype of MEN2A [12]. Codon 618 with p.Cys618Arg, p.Cys618Gly and p.Cys618Ser and to a lesser extent with codons p.Cys618Phe and p.Cys618Tyr, have been associated with the greatest rates of pheo [13–15]. Some studies revealed that the severity of pheo due to mutations in codons 609, 618 and 620 can be as aggressive as the one that is usually shown by the five amino acid substitutions at codon 634. However, the majority of the cases, demonstrated greatest expression for 634, followed in decreasing order by codons 618, 620 and 609 [13, 16, 17]. In recent reports, the spectrum of mutations identified in the *RET* proto-oncogene in patients with MTC has shifted from the ‘classical’ and most prevalent worldwide mutation at codon 634 in exon 11 to clinically less aggressive forms with mutations in exons 13–15 [18–20]. In the present study, we characterized clinically and molecularly Cypriot families with FMTC, MEN2A and MEN2B. The phenomenon of founder effect is not unusual in the population of Cyprus, and several recent reports have documented several founder mutations on the island. The recent evidence of a founder effect unveiled from the genetic population profile of certain endocrinopathies describes the past migration trends in Cyprus [21]. Therefore, we further investigated the possibility of our patients carrying a common allelic haplotype and identified a unique and identical haplotype in all patients carrying the missense p.Cys618Arg. Consequently, this common haplotype is the result of an ancestral mutation that has spread in the island of Cyprus due to a possible founder effect.

## Patients and methods

### Patients

Forty patients with MEN2 diagnoses (MTC with and without pheo) were screened for exons 10, 11, 13, 14, 15 and 16 of the *RET* proto-oncogene between January 2002 and September 2017. Except for one female patient of Russian descent the remaining 39 patients of the cohort were exclusively of Cypriot origin. Although defining and separating familial MTC from MEN2A and MEN2B has been challenging, the subjects were classified based on the symptoms presented so far. The specific clinical parameters used prior to sending for genetic testing included MTC, pheo, hyperparathyroidism and cutaneous lichen amyloidosis [1]. Informed consent for this study was obtained from all adult patients. Moreover, molecular testing for pre-symptomatic diagnosis of all minors was performed following informed consent obtained by the parents after appropriate genetic counselling and advice by a Pediatric Endocrinologist and a Nuclear Medicine Specialist.

### Oligonucleotides, PCR conditions and direct sequencing of the *RET* proto-oncogene

Genomic DNA was isolated from peripheral blood leukocytes using a kit from QIAGEN (QIAGEN, GmbH D-40724, Hilden, Germany). The primers and conditions for PCR amplification and direct sequencing of exons 10, 11, 13, 14, 15 and 16 were as described previously [15, 22].

### Genotyping with STR genetic markers flanking the *RET* proto-oncogene

In the present study, six STR markers flanking the *RET* proto-oncogene and according to GRCh38.p7 primary assembly reference sequence: NC_000010.11 were used. These genetic markers included STR1 (43207304–43207530 bp), STR2 (43664013–43664241 bp) and STR3 (43143426–43143640 bp) all located upstream of the *RET* proto-oncogene and the STR4 (43015181–43015361 bp), D10S469 (43445299–43445435 bp) and D10S681 (42897992–42897871 bp) all located downstream of the *RET* proto-oncogene. The forward primer of each genetic marker was labeled with 6-FAM fluorescent dye at 5′. The primer sequence pairs included STR1_F: 5′- CTTCAGTGCCATGACAGGAC-3′ & STR1_R: 5′-AGGAAACCTTTAGGTGTTTGGTG-3′; STR2_F: 5′- CTTTTCCAGACTCTCTCAAAGAGCAG-3′ & STR2_R: 5′-TATGGCAGACGTGGGTGCT-3′; STR3_F: 5′- GGAAAGTGGAATTGTGAATTGCTG- 3′ & STR3_R: 5′-GGCATAACACTTCACCTCTCAG-3′; STR4_F: 5′-GTTATTGTAGCCTCAGAGGC-3′ & STR4_R 5′-ACACTCTTCAAATCCATTCTGCG-3′; D10S469_F: 5′-GCAACAAGTGTGAGAGTCCAT-3′ & D10S469_R: 5′-GGATGTTCTGTCTCTCCACAGT-3′; D10S681_F: 5′- GACCAGAGGAAAGGCTAATGC-3′ & D10S681_R: 5′-GAAGCGGCCAGAACTTAGC- 3′. PCR was performed using 50 ng of genomic DNA, 1 × Amplitaq Gold Buffer (Applied Biosystems), 200 mΜ each dNTP (Sigma), 1,25 units of Amplitaq Gold DNA Polymerase (Applied Biosystems), 10 µM of each primer and double distilled water to a final volume of 25 μl. The amplification reaction was initialized at 95 °C for 10 min, followed by 35 cycles for 30 s at 95 °C, 60 s at 58 °C; 60 s at 72 °C and a final extension for 7 min at 72 °C. Each of the PCR products was diluted tenfold using ddH2O. Analysis of PCR products was performed by capillary electrophoresis on an ABI 3130xl Genetic Analyzer (Applied Biosystems) preceded by a denaturation step. A mix of 8.5 μl of HiDi™ formamide (Applied Biosystems) and 0.5 μl of GeneScan-600 LIZ size standard (Applied Biosystems) was added to 3 μl of diluted PCR product and denatured at 95 °C for 5 min. The mixture was immediately transferred on ice for 5 min and loaded for electrophoresis onto the ABI 3130xl genetic analyzer (Applied Biosystems). PCR products were visualized and analyzed with GeneMapper™ v4.1 Software (Applied Biosystems).

### Y-chromosome genotyping and haplogroup assignment

Since in Cyprus the gene pool is characterized by deep inbreeding and high degree of conservation, we decided to perform Y-STR haplotype analysis for all male patients carrying p.Cys618Arg. The methodology used was similar to the one of a recent paper [23]. This methodology allows the simultaneous determination of short tandem repeats (STRs) on 23 loci located on the Y-chromosome by the PowerPlex^®^ Y23 System (Promega). Subsequently, the 23 loci Y-STR haplotypes were input to the online Whit Athey’s Haplogroup Predictor tool [24] which generates probabilities for assignment to one of the major Y-DNA haplogroups.

## Results

### Clinical and genetic findings

*RET* mutations were identified in a total of 40 patients from 11 unrelated Cypriot families, one sporadic case of Cypriot descent and lastly one sporadic case of Russian descent. The cohort included 23 females (57.5%) and 17 males (42.5%). Among the 11 families, 9 probands (69.2%) were heterozygous for the missense p.Cys618Arg, one (7.7%) for the p.Cys634Phe, one (7.7%) for the somatic p.Glu632_Leu633del (delE632-L633) and two (15.4%) for the p.Met918Thr mutation. The mean age at MTC diagnosis in the group of patients carrying p.Cys618Arg was 36.8 ± 14.2 years (range 18–62 years). The age of pheo diagnosis in the same group ranged between 26 and 62 years and appeared simultaneously with MTC in 6/36 (16.7%) cases. The clinical and genetic findings of all patients and relatives diagnosed between 2002 and 2017 are summarized in Table 1.

**Table 1:** Clinical and genetic parameters of the MEN2 patients

Family	Gender	Mutation	Phenotype	Thyroidectomy	Age at diagnosis
A	M	p.Cys618Arg	MTC + pheo	Yes	26
	F	p.Cys618Arg	MTC	Yes	59
	M	p.Cys618Arg	MTC + pheo	Yes	30
	M	p.Cys618Arg	Asymptomatic*	Yes	–
	F	p.Cys618Arg	Asymptomatic*	Yes	–
	F	p.Cys618Arg	Asymptomatic*	Yes	–
B	M	p.Cys618Arg	MTC	Yes	34
	M	p.Cys618Arg	MTC	Yes	27
	F	p.Cys618Arg	Asymptomatic*	No	–
C	F	p.Cys618Arg	MTC	Yes	47
D	F	p.Cys618Arg	MTC + pheo	Yes	43
	M	p.Cys618Arg	Asymptomatic*	Yes	–
	F	p.Cys618Arg	MTC	Yes	27
	M	p.Cys618Arg	Asymptomatic*	No	–
E	F	p.Cys618Arg	MTC + pheo	Yes	41
	M	p.Cys618Arg	Asymptomatic*	Yes	–
	F	p.Cys618Arg	Asymptomatic*	Yes	–
F	F	p.Cys618Arg	MTC	Yes	21
	M	p.Cys618Arg	Asymptomatic*	No	–
G	F	p.Cys618Arg	MTC	Yes	61
H	M	p.Cys618Arg	MTC	Yes	18
	F	p.Cys618Arg	MTC	Yes	45
	M	p.Cys618Arg	Asymptomatic*	No	–
	M	p.Cys618Arg	Asymptomatic*	No	–
	F	p.Cys618Arg	Asymptomatic*	No	–
	F	p.Cys618Arg	MTC	Yes	19
	F	p.Cys618Arg	MTC + pheo	No	42
	M	p.Cys618Arg	Asymptomatic*	No	–
	F	p.Cys618Arg	MTC	No	19
	F	p.Cys618Arg	MTC	No	43
	F	p.Cys618Arg	MTC + Breast Cancer	Yes	58
	F	p.Cys618Arg	MTC	Yes	28
	F	p.Cys618Arg	MTC	Yes	29
	M	p.Cys618Arg	MTC	No	30
I	F	p.Cys618Arg	MTC + pheo	Yes	62
	M	p.Cys618Arg	Asymptomatic*	No	–
K	M	delE632-L633	MTC	Yes	49
L	F	p.Cys634Phe	MTC + pheo	Yes	38
M	M	p.Met918Thr	MTC-pheo-(MEN2B)	Yes	25
N	F	p.Met918Thr	MTC − (MEN2B)	Yes	15

Letters A–I indicate the families carrying the p.Cys618Arg. Letters K–L indicate sporadic cases with other MEN2A causing mutations. Letters M–N indicate sporadic cases with the MEN2B p.Met918Thr mutation. *Asymptomatic to the last day of evaluation

**Fig. 1:**
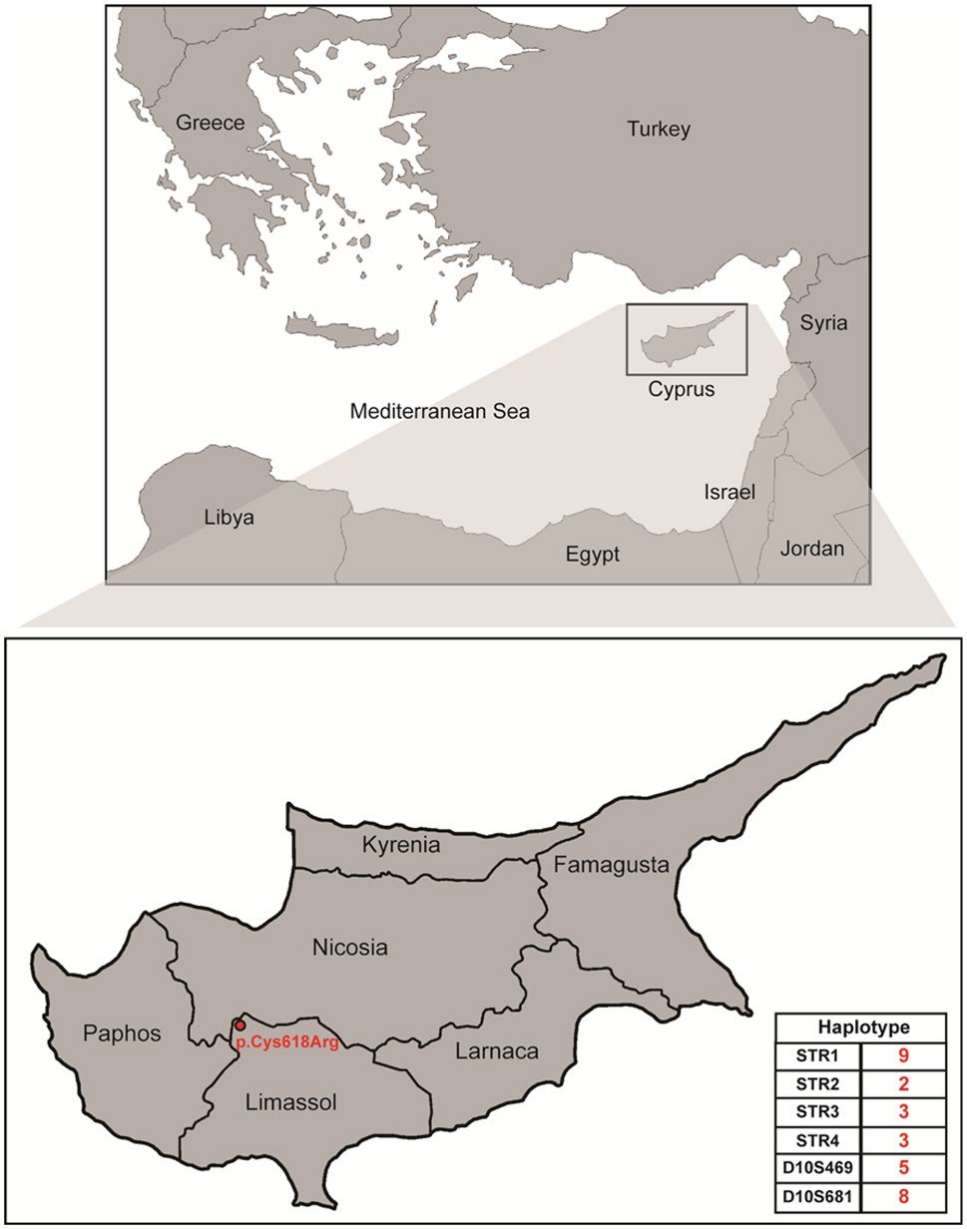
Geographical representation of the common origin of the five out of nine families carrying the p.Cys618Arg mutation. The island of Cyprus located in the eastern Mediterranean Sea. The village is located at the north-western end of the Limassol province and is indicated with red colour. The common haplotype which is segregated with the p.Cys618Arg mutation is demonstrated at the right bottom of the figure

Screening of family members from all nine MEN2A families carrying the p.Cys618Arg mutation showed the presence of the same genetic defect in thirty-six individuals of various ages, 24 of whom underwent prophylactic thyroidectomy (Table 1). Family histories of five out of nine MEN2A families with the p.Cys618Arg mutation reveal that they originated from the same village at the north-western end of the Limassol province in Cyprus (Fig. 1).

Only eight out of the total forty patients with MEN2 were clinically reported as having both MTC and pheo. Six of these patients belonged to the group identified with the missense p.Cys618Arg mutation in exon 10 of the *RET* proto-oncogene. Unfortunately, two of the patients with MEN2A carrying p.Cys618Arg passed away and no clinical follow-up as far as the development of pheo and their clinical status was available. Thus far, from the remaining MEN2A patients carrying the same p.Cys618Arg, sixteen manifested only MTC and fourteen were asymptomatic (Table 1). Therefore, a strong manifestation of MTC with a wide range of ages was observed in these families (Table 1).

The patients identified with the sporadic p.Cys634Phe and the somatic p.Glu632_Leu633del (delE632-L633) mutation also causing MEN2A only had MTC at the time of diagnosis. Lastly, two discrete patients were both identified with the severe p.Met918Thr MEN2B causing mutation. The first patient is a male who unfortunately passed away at the age of 39 and who underwent total thyroidectomy when he was clinically diagnosed with MTC and pheo at the age of 25. The second patient is currently a 16-year-old female who up to now exhibited only MTC. Recently, a year after her initial diagnosis she underwent total thyroidectomy with central node dissection (Table 1).

### Haplotype analysis of families with the *RET* proto-oncogene p.Cys618Arg missense mutation

**Fig. 2:**
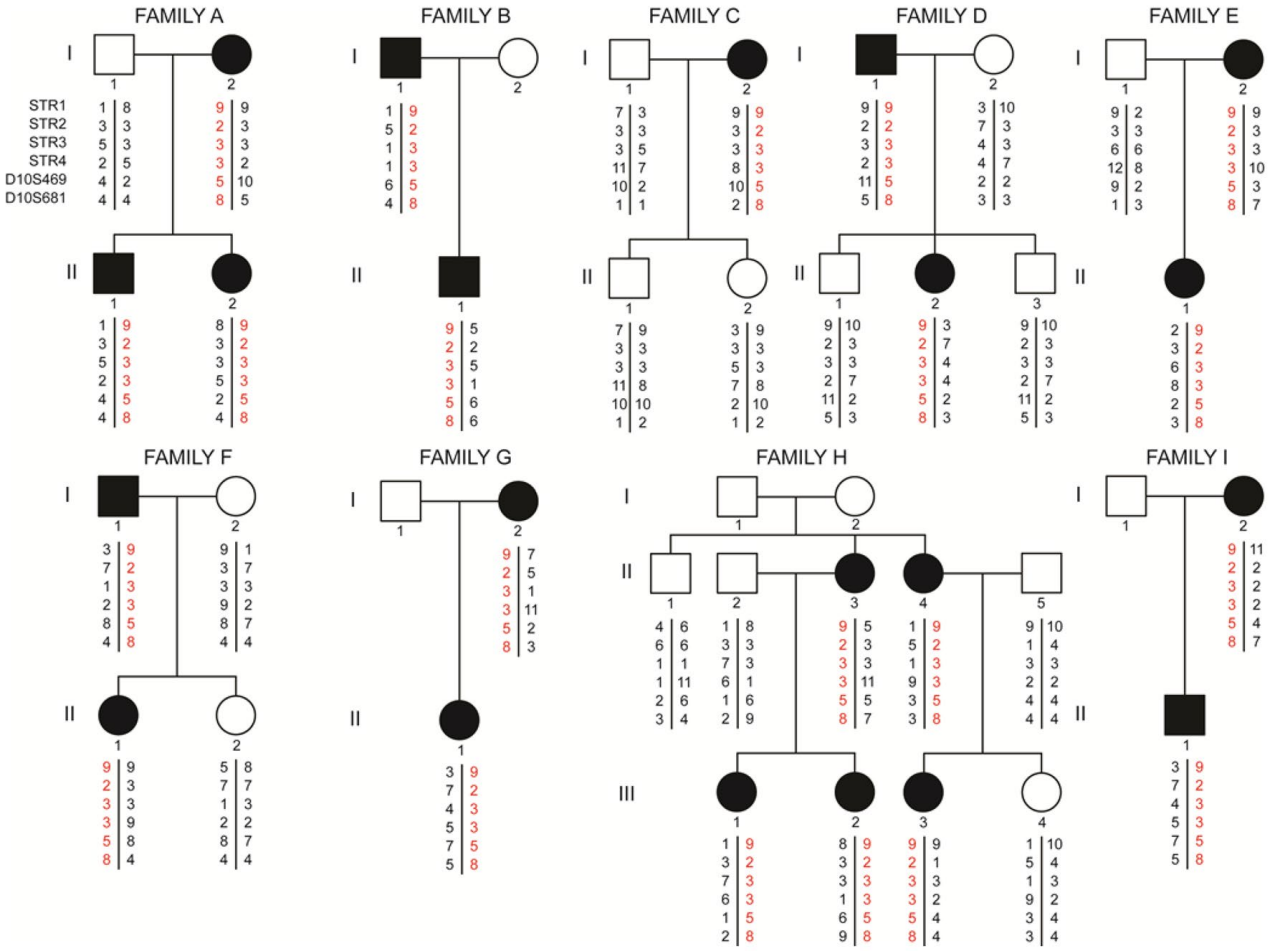
Pedigree and disease-haplotype segregation of families with the *RET* proto-oncogene p.Cys618Arg missense mutation. Blackened symbols represent affected individuals with MEN2A phenotype. White symbols represent individuals with a normal phenotype. Circles and squares indicate females and males, respectively. Red characters represent the mutant haplotype which segregates with the p.Cys618Arg missense mutation

In the present study, a common haplotype was demonstrated to be present in a cohort of nine and apparently non-related Cypriot MEN2A families that all carried the same missense p.Cys618Arg (Fig. 2). This specific haplotype was not found in the rest of the examined Cypriot MEN2 probands and their families identified with other than the specific p.Cys618Arg defects (Table 1).

### Y-haplogroup assignment

A total of seven different major Y-haplogroups (G2a, J1, R1b, R1a, I2a, E1b1a, J2b) were predicted amongst the 10 typed male Cypriot patients all carrying p.Cys618Arg mutation. The most frequent Y-haplogroup was G2a (40%) and was followed by J1, R1b, R1a, I2a, E1b1a and J2b at 10%, each.

## Discussion

The distribution of genetic defects in our cohort differed from that of other populations in the context that the *RET* p.Cys618Arg mutation was found to be the most prevalent (69.2%). According to the latest ATA Management Guidelines, mutations at RET codon C618 are categorized as ATA moderate (MOD) level and carry a lower risk for aggressive MTC (ATA levels are categorized as moderate (MOD), high (H) and highest risk (HST) [1].

The risk of MTC has been stratified in three types according to the mutations of the *RET* proto-oncogene. Children with MEN2B due to defects in codon 883, 918 and 922 have the greatest risk of aggressive MTC (HST) and ought to undergo a total thyroidectomy with central node dissection, within the first 6 months [1]. Children bearing any of the RET codons 611, 618, 620 are prone to MOD risk of MTC, while those bearing 634 the majority of times exhibit a high (H) risk. Thus, a total thyroidectomy should be carried out before the age of 5 years. Children with defects in the RET codons 609, 768, 790,791, 804 and 891 have in general a less aggressive MTC and thyroidectomy may be performed at a later stage [1, 25].

In 85% of MEN2A patients, RET codon 634 (p.Cys634Arg) has been reported as the most prevalent in European and non-European families [18, 20, 26]. Several other smaller and larger multicenter studies mainly from groups around Europe demonstrated codon 533 as the prevailing in Greece [27], 611 in Portugal and Denmark [28, 29], 618 in Cyprus [15], 790 in Germany [18] and 804 and 891 in Italy [19].

It is estimated that more than 50% of familial MTC cases occur in patients with defects in codon 618. According to the most recent consensus guidelines, aberrations in codon 618 are classified as moderate (MOD) for MTC for which prophylactic thyroidectomy is recommended prior to the age of 5 years [1, 30]. All nine probands of the current study carrying the missense p.Cys618Arg and several of their close relatives also identified with the specific defect underwent prophylactic thyroidectomy (Table 1). Pheo is a distinct constituent of MEN2 syndrome and as reported it occurs in 15–20% of MEN2 patients, depending on age, follow-up time and type of *RET* mutation [10, 17, 31]. The coexistence of MTC and pheo usually establishes the diagnosis of either MEN2A or MEN2B [1] and in the present study, it was observed in 8/40 patients identified with *RET* mutations. Generally, pheo penetrance and age of diagnosis highly correlate with MTC aggressiveness based on the *RET* mutation status. The coexistence of pheo and MTC was only observed in one of the two patients with MEN2B due to p.Met918Thr, a 25-year-old male who underwent thyroidectomy. The other MEN2B female patient is currently 16 years old and up to now only manifested MTC. Since penetrance of pheo progressively increases with age an at risk-patient such as the young female of the present study identified with the MEN2B as a result of p.Met918Thr, recently underwent total thyroidectomy with central node dissection. MTC is a calcitonin making tumor of the parafollicular cells of the thyroid gland, which is preceded by multifocal C cell hyperplasia. The timing of the progression of MTC in relation to the incidence of pheo is variable and may take many years and not all mutations have the same effect on how aggressive the MTC is [32]. In general, *RET* mutations occurring in the extracellular cysteine domains (e.g., C611, C618, C620) of the gene have weaker transforming ability as a result of being more distant from the cell membrane when compared to those located close to the cell membrane such as p.Met918Thr [30, 33]. This phenomenon explains the varied ages from 15 to 62 years at which the probands and their relatives were diagnosed. The aggressiveness of MTC correlates with pheo penetrance and age at diagnosis and is usually associated with *RET* mutation codon status [34]. Prevention or therapy of MTC is successfully achieved with thyroidectomy once the diagnosis is made or before the age of likely malignant progression [1, 34]. On the basis of our genetic screening and clinical evaluation performed during a follow-up of 1–15 years, we report a higher prevalence of Familial MTC in the group of patients identified with the same missense p.Cys618Arg mutation.

In the present study, the common haplotype was exclusively found in the cohort of Cypriot MEN2A families carrying the missense p.Cys618Arg mutation. Based on the fact that this specific haplotype was not found in the remaining Cypriot MEN2 probands identified with other than the specific p.Cys618Arg mutation, speculates that it is the result of an ancestral mutation that has spread in the island of Cyprus due to a possible founder effect (Fig. 2).

In addition to the probable founder effect suggested here, similar phenomena have been projected for a number of other mutations or diseases among the population of Cyprus such as the predominance of the IVS1-2A > G mutation in the *5α Steroid Reductase type 2* (*SRD5A2*) gene in patients with 5 Alpha Reductase deficiency [35], the frequency of 1:7–1:10 for Friedreich ataxia [36] and the reported novel *COL4A4* gene mutation p.Gly871Cys in a cohort of Greek-Cypriot families with thin membrane nephropathy and focal segmental glomerulosclerosis [37].

As reported in the early seventeenth century, in a village listed as property of the Venetian Government’ at the north-western end of the Limassol province in Cyprus, an infectious disease plunged this village and nearby settlements (Fig. 1). We speculate that the reported disease of that time was the result of a founder mutation such as p.Cys618Arg. Based on the genetic data produced in the present study, we have now speculation that this founder mutation was introduced to the locals by an invader or a settler during the Venetian era between 1489 and 1570 or prior to that period during the Crusades and the Lusignan Period 1191–1489. To investigate whether a common Y-haplogroup could be demonstrated in male individuals harboring the p.Cys618Arg mutation, we extended our study by performing a Y-chromosome analysis on male patients identified with the abovementioned mutation. In the cohort of male patients heterozygous for the p.Cys618Arg mutation, seven different major Y-haplogroups (G2a, J1, R1b, R1a, I2a, E1b1a, J2b) were found which were previously identified in Y-chromosomal studies of the Cypriot population [23, 38]. The different observations regarding the inheritance of the p.Cys618Arg mutation (suggesting a founder effect) and the genetic variety of Y-haplogroups in these individuals from Cyprus may indicate dissimilarities in the inheritance patterns between autosomal and Y-chromosome markers due to genetic drift, gene flow bias and other population level events.

In conclusion, the distribution of *RET* proto-oncogene mutations in Cyprus differs from that of other populations. The missense mutation p.Cys618Arg in codon 10 is by far the most prevalent mutation followed by other reported mutations of variable clinical significance. Additionally, the present study is the most extensive study ever accomplished addressing the ancestral *RET* proto-oncogene p.Cys618Arg mutation origin and including a large number of Cypriot families. The provided molecular evidence speculates that the most prevalent p.Cys618Arg mutation could be the result of an ancestral mutation that has spread in the island of Cyprus due to a possible founder effect.
